# The "Hospital Penny Fund."

**Published:** 1904-05-14

**Authors:** 


### THE "HOSPITAL PENNY FUND."

HOSPITAL MANAGERS throughout the country are indebted to *Truth* for calling attention to a scheme for collecting penny subscriptions for the various hospitals throughout the United Kingdom to be known as the "Hospital Penny Fund." The documents which have been circulated are marked "private and confidential," and any hospital manager who reads them will probably feel that in effect the scheme is a private venture for raising money in penny subscriptions. At least 25 per cent. of the gross receipts is to be retained by the promoters to provide prizes, working expenses, and the remuneration of the controller and manager, etc., a sufficient reason in itself to cause every hospital representative not only to decline to be a party to it, but to take active steps to put an end to it. It would be open to condemnation as a serious proposal if the cost was limited to 25 per cent. of the gross receipts, but the promoters are careful to point out that it may be found "impossible" to satisfy all their claims by a deduction of 25 per cent., in which case the percentage may be increased.

In brief, the proposal is to issue stamps containing pictures of particular hospitals, and each such hospital is asked to purchase a number of books containing these stamps by handing to the promoters a cheque for 25 per cent. of the face value of all stamps thus supplied. The hospital can then sell as many of these stamps as possible, taking all the risk of sale, and so may almost certainly find themselves out of pocket by the transaction. Seeing that at present any hospital may obtain from the King's Hospital Fund stamps of various face values free of cost, which they can sell without risk of loss, the proposal on the face of it is ridiculous.

The scheme further involves most of the objections which attach to a lottery. In order to bring in the general public, £7,000 is to be offered in cash prizes of from £3,000 to £5 each in value, together with 5,000 watches to be given away to those who enter the competition. If this prize money was to be given by the promoters out of their own pockets the scheme would be very objectionable, but when it is seen that the hospitals and the public are asked to place this money in the hands of the promoters for the purposes of the scheme, it stands condemned on its merits. Any reputable charity in these days which spent every year 25 per cent. in raising its income would soon become discredited and fail before long to receive the support of the public. The promoters have been trying in various ways since 1902 to secure the names and assistance of persons of repute and public position in support of this movement. So far they have failed and they deserve to fail. We call attention to the matter to-day because an attempt is being made to induce members of the House of Peers and others to associate themselves with the promoters by joining a committee which they are attempting to form to aid them in introducing this private adventure scheme to the public. It is within our knowledge that those who know most of the subject have steadily refused to have anything to do with the Hospital Penny Fund, and we are confident that anyone who takes the trouble to make full inquiries and who has the interests of the hospitals at heart will have nothing to do with an enterprise of this character.

In the documents which are being circulated in support of this scheme a somewhat free use has been made of the name of the King's Hospital Fund. We cannot believe that permission has been granted to connect the King's Fund with this scheme, and we suggest that anybody who is approached by the promoters should communicate with the honorary secretaries of the King's Fund, 81 Cheapside, E.C., and so satisfy himself on this point.

